# Elevated Sodium Pump α3 Subunit Expression Promotes Colorectal Liver Metastasis *via* the p53-PTEN/IGFBP3-AKT-mTOR Axis

**DOI:** 10.3389/fonc.2021.743824

**Published:** 2021-11-12

**Authors:** Di Wu, Hong-Qiang Yu, Hao-Jun Xiong, Yu-Jun Zhang, Xiao-Tong Lin, Jie Zhang, Wu Wu, Teng Wang, Xiao-Yu Liu, Chuan-Ming Xie

**Affiliations:** ^1^ Key Laboratory of Hepatobiliary and Pancreatic Surgery, Institute of Hepatobiliary Surgery, Southwest Hospital, Third Military Medical University (Army Medical University), Chongqing, China; ^2^ Department of Hepatobiliary Surgery, The Second Affiliated Hospital of Chongqing Medical University, Chongqing, China; ^3^ Department of Oncology, Affiliated Hospital of Jiangnan University, Wuxi, China; ^4^ School of Medicine, Southern University of Science and Technology, Shenzhen, China

**Keywords:** colorectal cancer, metastasis, Na+/K+-ATPase, mTOR, p53, PTEN (phosphatase and tensin homolog deleted on chromosome 10), IGFBP3

## Abstract

The sodium pump α3 subunit is associated with colorectal liver metastasis. However, the underlying mechanism involved in this effect is not yet known. In this study, we found that the expression levels of the sodium pump α3 subunit were positively associated with metastasis in colorectal cancer (CRC). Knockdown of the α3 subunit or inhibition of the sodium pump could significantly inhibit the migration of colorectal cancer cells, whereas overexpression of the α3 subunit promoted colorectal cancer cell migration. Mechanistically, the α3 subunit decreased p53 expression, which subsequently downregulated PTEN/IGFBP3 and activated mTOR, leading to the promotion of colorectal cancer cell metastasis. Reciprocally, knockdown of the α3 subunit or inhibition of the sodium pump dramatically blocked this effect *in vitro* and *in vivo via* the downregulation of mTOR activity. Furthermore, a positive correlation between α3 subunit expression and mTOR activity was observed in an aggressive CRC subtype. **Conclusions:** Elevated expression of the sodium pump α3 subunit promotes CRC liver metastasis *via* the PTEN/IGFBP3-mediated mTOR pathway, suggesting that sodium pump α3 could represent a critical prognostic marker and/or therapeutic target for this disease.

## Introduction

Colorectal cancer (CRC) is one of the most common malignancies and the third most common cause of cancer-related mortality in the world (1, 2). Metastasis is the major cause of poor outcomes in CRC patients. The liver is frequently the most common site of metastasis in more than 50% of patients with CRC (3, 4). Surgical resection remains the only potential curative therapeutic option, but less than 20% of patients are eligible for surgical resection because they do not meet the criteria (5). The underlying mechanisms of CRC liver metastasis are not well understood, which limits the efficacy of chemotherapy.

Na+/K+-translocating adenosine triphosphatase (Na+/K+-ATPase, also named sodium pump) belongs to the P-type ATPase family that transports sodium and potassium across the plasma membrane (6, 7). The sodium pump is an oligomeric protein composed of α subunits, β subunits and FXYD proteins. Humans express four isoforms of α subunits, three isoforms of β subunits and seven isoforms of FXYD proteins (8–10). These four α isoforms are expressed in a tissue-dependent manner in mammalian cells. α1 is expressed in all cells, while α2 is predominantly expressed in the heart, skeletal tissue, smooth muscle and brain (11–13). α3 is detected in neurons and heart. α4 is primarily expressed in testis (11, 12, 14). Numerous studies have indicated that sodium pumps are abnormally expressed in various cancers, including CRC, lung cancer, breast cancer, and liver cancer (8, 15–17). The sodium pump is a main target of cardiac glycosides, including bufalin and digoxin (18). Cardiac glycosides, which are therapeutic agents for heart failure treatment, are natural compounds derived from plants and animals, such as *Digitalis lanata* and *Bufo arenarum* (19, 20). These compounds display higher selectivity for the α2 and α3 isoforms over the α1 isoform (21). Previous studies by our group and others have demonstrated that the α3 subunit is the most highly expressed subunit in colorectal cancer (22, 23). Taken together, cardiac glycosides may be potential therapeutic drugs for CRC patients with high α3 expression.

The tumor suppressor protein p53 plays an important role in the prevention of carcinogenesis. p53 regulates cell proliferation and metastasis *via* regulation of its downstream targets PTEN, IGFBP3, TSC2, AMPK1, or PHLDA3 (24, 25). PTEN, TSC2 and AMPK1 are negative regulators of mTOR activation. IGFBP3 and PHLDA3 inactivate mTOR *via* PI3K/AKT (26, 27). mTOR is a critical pathway related to metastasis (28). Current studies have reported that more than 40-50% of CRC patients have wild-type p53 (29, 30). In this study, we found that elevated expression of sodium pump α3 promoted CRC liver metastasis *via* downregulation of p53-PTEN/IGFBP3 and upregulation of mTOR activity. Targeting sodium pumps with the cardiac glycoside bufalin significantly attenuated CRC liver metastasis.

## Materials and Methods

### Reagents and Antibodies

Bufalin (S7821) was obtained from Selleck Industries LLC (Shanghai, China). Fetal bovine serum (FBS; 16000-044), Dulbecco’s modified Eagle’s medium DMEM (C11995500bt), McCoy’s 5A medium (16600082) and L15 medium (11415064) were purchased from Gibco (Thermo Fisher Scientific, Inc., Waltham, MA, USA). Anti-mouse IgG-HRP (7076), anti-rabbit IgG-HRP (7074), anti-mTOR (2972S), anti-p-mTOR [(Ser2448) 5536S], anti-p-AKT [(Ser473) 4060S], anti-PTEN (9188S), anti-p-S6K [(Thr389) 9234S], anti-E-cadherin (14472S), and anti-p-4EBP1 [(Thr37/46) 2855S] antibodies were obtained from Cell Signaling Technology, Inc. (Danvers, MA, USA). Anti-vimentin (10366-I-AP), anti-IGFBP3 (10189-2-AP), anti-p53 (10442-I-AP), anti-sodium pump α3 (10868-I-AP), and anti-β-actin (60008-I-Ig) antibodies were obtained from Proteintech (Wuhan, China). Anti-mouse Flag (F1804) antibody was obtained from Sigma.

### Human Subjects

In total, 91 human CRC tissue specimens and paired adjacent paraffin tissue specimens were obtained from the Affiliated Hospital of Jiangnan University, Jiangsu, P.R. China. The tissue samples were used for immunohistochemical (IHC) analyses. This study was approved by the Ethics Committee of Affiliated Hospital of Jiangnan University, and all of the patients provided informed consent. Detailed clinical and pathological data were obtained from each patient.

### Cell Culture

Human colon cancer HCT-116, HT29, SW620, and SW480 cells and normal colon CCD841 cells were purchased from American Type Culture Collection (ATCC). HCT-116 cells were cultured in McCoy’s 5 A medium, HT29 and CCD841 cells were cultured in Dulbecco’s modified Eagle’s medium (DMEM), and SW620 and SW480 cells were cultured in L15 medium. All the experiments were carried out in medium containing 10% FBS, 100 U/ml penicillin and 100 mg/ml streptomycin (Invitrogen) at 37°C in 5% CO_2_.

### Reverse Transcription PCR

Total RNA was extracted using RNAiso plus (9109, Takara). Complementary DNA was synthesized using the Prime Script™ RT Reagent Kit with gDNA Eraser (RR047A, Takara) according to the manufacturer’s instructions. Quantitative real-time PCR (qPCR) was performed using TB Green™ Premix Ex Taq™ II (Tli RNaseH Plus; RR820A, Takara). The PCR cycle parameters were as follows: 95°C for 30 s, followed by 40 cycles at 95°C for 5 s and 60°C for 1 min. The results were obtained with CFX96™ Real-time System 3.0 software (Applied Bio-Rad) and further analyzed by the 2–ΔΔct method. Anti-β-actin was used as a loading control. The results are shown as the fold-change relative to the control group. The primer-specific sequences were as follows:

α1- Forward: 5’- AGTACACGGCAGTGATCTAAAGG-3’;
α1- Reverse: 5’-CAGTCACAGCCACGATAGCAC-3’;
α2- Forward: 5’-GGAGATGCAAGATGCCTTTCA3’;
α2- Reverse: 5’-GCTCATCCGTGTCGAATTTGA3’;
α3- Forward: 5’- GACCTCATTTGACAAGAGTTCGC-3’;
α3-Reverse: 5’- GGGCAGACTCAGACGCATC-3’;
β1-Forward: 5’-CGGGAAAGCCAAGGAGGAG-3’
β1- Reverse: 5’-TCTGTGTTAATCCTGGCGGG-3’
β2-Forward: 5’-CGTGCTTTTGGGTGTGTGGA-3’
β2- Reverse: 5’-AGAAGAGGAGGATAAAGGCCCA-3’
β3-Forward: 5’-TCGAGTACTCCCCGTAACGA-3’
β3- Reverse: 5’-GAGCAAGATCAAACCCCAGC-3’
PTEN-Forward: 5’-CTCAGCCGTTACCTGTGTGT-3’;
PTEN-Reverse: 5’-AGGTTTCCTCTGGTCCTGGT-3’;
TSC2-Forward:5’-TACGAGTGCAACCTGGTGTC-3’;
TSC2-Reverse:5’-GAGGCCATATTTGCGTGCAG-3’;
Ampk1-Forward:5’-AAAGTCGGCGTCTGTTCCAA-3’;
Ampk1-Reverse:5’-GGGCCTGCATACAATCTTCCT-3’;
IGFBP3-Forward:5’-TGTGGCCATGACTGAGGAAA-3’;
IGFBP3-Reverse:5’-TGCCGACCTTCTTGGGTTT-3’;
PHDLA3-Forward:5’-CAGTAGGGGCTGAGCATGAA-3’;
PHDLA3-Reverse: 5’-GCAGTCTGCAGAACCCAGAA-3’;
β-actin-Forward: 5’GAGAAAATCTGGCACCACACC-3’; and
β-actin-Reverse: 5’GGATAGCACAGCCTGGATAGCAA -3’;

### Western Blotting

The procedure for Western blotting analysis was described in a previous report (31). Briefly, cells were lysed with lysis buffer (20 mM Tris-HCl (pH 7.4), 150 mM NaCl, 1 mM EDTA, 1% Triton X-100, 2.5 mM sodium pyrophosphate, 1 mM DTT, 1 mM sodium orthovanadate, 1 lg/ml leupeptin, 1 mM phenylmethylsulfonyl fluoride) on ice for 1 h. The protein concentration was determined with the Coomassie Protein Assay reagent. Equal amounts of protein extracts (10-50 μg) were separated by 10-12% sodium dodecyl sulfate (SDS)-polyacrylamide gel electrophoresis (PAGE) and transferred to nitrocellulose filter (NC) membranes. The membranes were blocked in 5% nonfat milk in Tris-buffered saline containing 0.1% Tween 20 (TBST) for 2 h at room temperature and then incubated with primary antibodies (1:1000) overnight at 4°C. After washing three times with TBST, the membrane was incubated with a peroxidase-conjugated secondary antibody for 1 h, developed with ECL reagent and analyzed by densitometric analyses using the Bio-Rad Imaging System. The intensity of each band was quantified using ImageJ and normalized to β-Actin. The data are expressed as relative changes.

### Small Interfering RNA Transfection

The cells were transfected with a negative control siRNA or siRNA targeting α3 or p53 at a 90 nM concentration for each siRNA duplex in Opti-MEM *via* Lipofectamine 2000 according to the manufacturer’s protocol. The siRNA oligos were purchased from Shanghai Gene Pharma Co., Ltd. (Shanghai, China), and their sequences were as follows: siα3#1, 5’-ACGACAACCGAUACCUGCUGGUGAU-3’; siα3#2, 5’-GCGUGCUUGGUUUCUGCCAUUAUUA-3’; sip53#1, 5’-GAAAUUUGCGUGUGGAGUATT-3’; sip53#2, 5’-GACUCCAGUGGUAAUCUACTT-3’; a nontarget siRNA (siControl), 5’-UCUACGAGGCACGAGACUU-3’.

### Transwell Assay

Cell migration and invasion were evaluated by Transwell assay. Briefly, the cells were adjusted to a concentration of approximately 1×10^5^ cells/ml in serum-free medium, 200 μl of the cell suspension was added to the upper chamber with (for cell invasion) or without (for cell migration) matrigel, and 600 μl of DMEM containing 20% FBS was added to the lower chamber. The cells were incubated in the Transwells for 24 or 48 h. A cotton-tipped applicator was used to carefully remove the cells that had not migrated/invaded from the top of the membrane. The membrane was washed twice with PBS, fixed with 4% formaldehyde for 15 min, and then stained with 0.5% crystal violet. The migrated cells were observed and photographed under a light microscope.

### Immunohistochemistry Staining

The procedure for immunohistochemistry staining was described in a previous report (32). Paraffin-embedded tissues were sectioned to a thickness of 3 μm. After routine deparaffinization, rehydration, blocking with hydrogen peroxide, and tissue antigen retrieval with a microwave, the samples were incubated with rabbit anti-α3 polyclonal antibody (sc-365744, 1:300, Santa Cruz Biotechnology) or rabbit anti-p-4EBP1 antibody (#2855, 1:300, Cell Signaling Technology) overnight at 4°C. The slides were stained with secondary antibody, incubated with DAB (ZSGB-BIO, China), and then counterstained with hematoxylin. The stained slides were evaluated independently by 2 investigators who were unaware of the clinical parameters.

### Tumor Orthotopic Xenograft Mouse Model

To investigate the role of sodium pump α3 on the metastatic effect of CRC *in vivo*, we established a liver metastasis model using 8-week-old female, specific pathogen-free BALB/c nude mice. All the mice were housed in a specific pathogen-free environment in the Animal Laboratory Unit. HCT-116 cells (5×10^6^ cells in 100 μl serum-free DMEM medium) were subcutaneously inoculated into the right flank of the nude mice. After two weeks, the tumors were isolated and cut into 2-3 mm^2^ pieces and implanted into the cecum of 8-week-old female nude mice to generate an orthotopic xenograft mouse model. For the α3 decreased expression model, HCT-116 cells stably expressing shRNA-control or shRNA α3 were used. For the drug treatment model, HCT-116 cells were used to generate an orthotopic xenograft mouse model, and the mice were treated with the sodium pump inhibitor bufalin (1.5 mg/kg) every other day for six weeks. Finally, the mice were euthanized, and liver samples were collected. The metastatic nodules were counted. All the animal experiments were approved by the Institutional Animal Care and Use Committee of Third Military Medical University.

### Statistical Analysis

The Pearson χ2 test or Fisher’s exact test was used to analyze the correlation between α3 expression and the clinicopathological features of the CRC patients. OS was estimated using the Kaplan-Meier method. Student’s t-test was used for comparison of two groups or one-way analysis of variance (ANOVA) for comparison of more than two groups followed by Tukey’s multiple comparison test. For multiple testing, a Bonferroni *post hoc* test of p-values was used using GraphPad Prism 6 (GraphPad, Inc., San Diego, CA, USA). The data are expressed as the mean ± SEM of at least three independent experiments. A p value <0.05 was considered to be statistically significant.

## Results

### High Expression of α3 Is Associated With Metastasis of CRC

**Figure 1:**
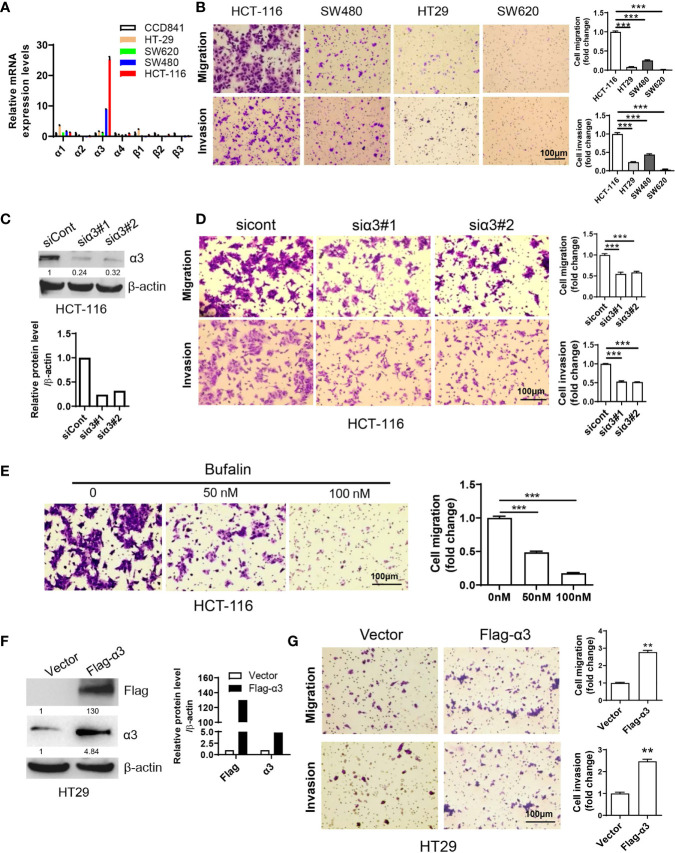
Sodium pump α3 subunit promotes CRC cell migration. **(A)** The relative mRNA expression levels of sodium pump α and β subunits in colon cancer HCT-116, HT29, SW620 and SW480 cells and normal colon CCD841 cells were examined by qPCR. β-actin was used as an endogenous control. **(B)** Transwell assays were used to test the migration and invasion ability of HCT-116, HT29, SW480 and SW620 cells. Scale bar, 100 μm. **(C, D)** HCT-116 cells were transfected with non-targeting siRNA (siCont) or siRNA targeting α3 (siα3) for 48 h. A portion of cells was harvested for Western blotting **(C)**. The other portion was plated into Transwell plates overnight, and migration and invasion were quantified after crystal violet (0.5% w/v) staining **(D)**. n=3-4, biological replicates. **(E)** HCT-116 cells were treated with bufalin (0, 50, or 100 nM) for 48 h and then plated into Transwell plates overnight. The migrated cells were quantified after crystal violet (0.5% w/v) staining. n=3-4, biological replicates. **(F, G)** HT29 cells were transfected with empty vector or Flag-α3 plasmids for 48 h. A portion of cells was harvested for Western blotting **(F)**. The other portion was plated into Transwell plates overnight, and migration and invasion were quantified after crystal violet (0.5% w/v) staining **(G)**. n=3-4, biological replicates. Scale bar, 100 µm. The error bars represent SEM from two or three independent experiments. One-way ANOVA with Tukey’s multiple comparisons test was used **(B**, **D, E)**. Two-way ANOVA with Bonferroni’s multiple comparisons test was used **(A)**. Unpaired student t-test was used in **(G)**, **p ≤ 0.01, ***p ≤ 0.001.

To investigate the function of sodium pumps in CRC, we examined the expression levels of the α and β isoforms of sodium pumps in CRC cell lines by qPCR. We found that α3 expression levels in HCT-116 cells were much higher than those in other CRC cell lines (HT29, SW620 and SW480 cells) and the normal colon cell line CCD841 (**Figure 1A**). Consistent with this finding, the migration and invasion rates were significantly higher in the HCT-116 cell line than in other colorectal cancer cell lines (**Figure 1B**). We wanted to know whether the expression level of α3 was associated with metastasis in CRC. We next knocked down α3 in HCT-116 cells with high α3 expression and found that knockdown of α3 could significantly inhibit cell migration and invasion (**Figures 1C, D**). Consistent with this finding, the sodium pump inhibitor bufalin also blocked the migration of HCT-116 cells in a dose-dependent manner (**Figure 1E**). Reciprocally, we transfected HT29 cells with empty vector or pcDNA3.1-α3(**Supplementary Information**). Overexpression of α3 in HT29 cells with low α3 expression dramatically promoted cell migration and invasion (**Figures 1F, G**). Taken together, the expression levels of the sodium pump α3 subunit are positively associated with metastasis of CRC.

### The α3-p53-PTEN/IGFBP3-mTOR Axis Is Associated With Metastasis of CRC

**Figure 2:**
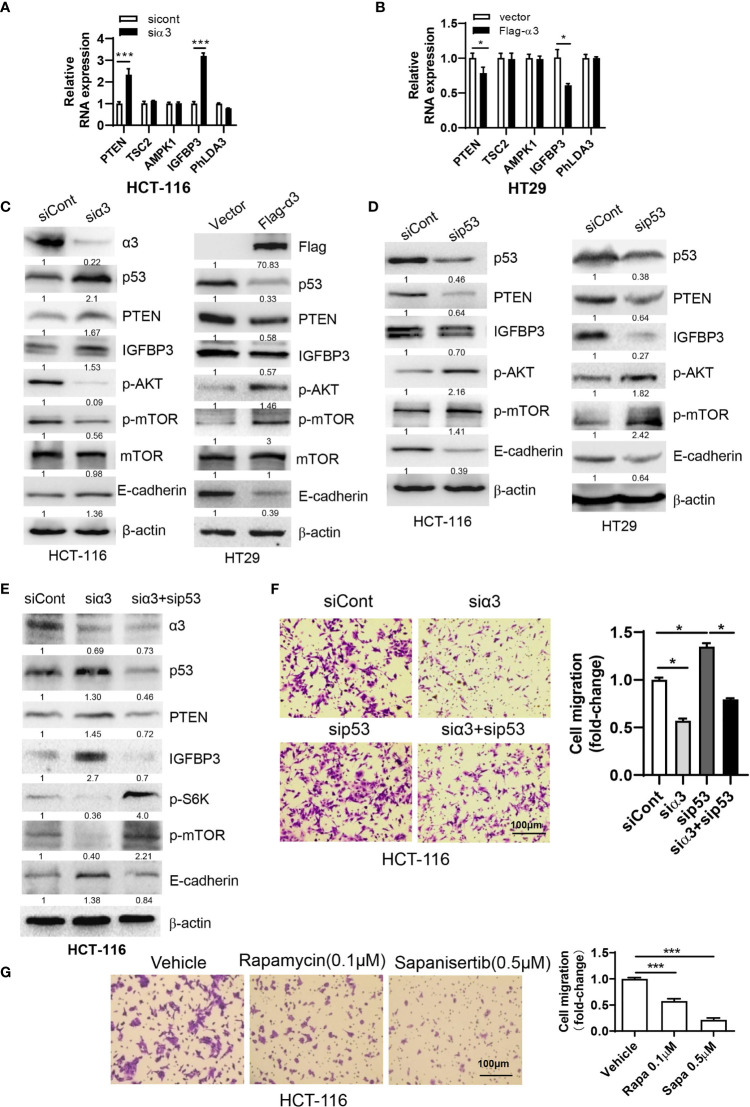
α3 promotes CRC cell migration *via* p53-PTEN/IGFBP3-mediated mTOR. **(A, B)** The mRNA expression levels of p53 downstream targets PTEN, TSC2, AMPK1, IGFBP3 and PhLDA3 were analyzed by qPCR in HCT-116 cells after knockdown of α3 for 48 h **(A)** or in HT29 cells upon overexpression of α3 for 48 h **(B)**. **(C, D)** HCT-116 or HT29 cells were transfected with the indicated plasmids or siRNAs for 48 h. The protein levels of α3, P53, PTEN, IGFBP3, p-AKT, mTOR, p-mTOR, and E-cadherin were analyzed by Western blotting. **(E, F)** HCT-116 cells were transfected with the indicated siRNAs for 48 h. A portion of cells was harvested for Western blotting **(E)**. The other portions were replated into Transwell plates overnight and stained with crystal violet (0.5% w/v) **(F)**. **(G)** HCT-116 cells were treated with the mTOR inhibitors rapamycin (Rapa, 0.1 µM) and sapanisertib (Sapa, 0.5 µM) for 48 h, followed by crystal violet (0.5% w/v) staining to test cell migration. Scale bar, 100 µm. The data are expressed as the mean ± SEM (n=3). Two-way ANOVA with Bonferroni’s multiple comparisons test was used in **(A, B)**. One-way ANOVA with Tukey’s multiple comparisons test was used in **(F, G)**. *p ≤ 0.05, ***p ≤ 0.001.

Previous studies indicated that p53 played a critical role in the negative regulation of metastasis (33, 34). As HCT-116 cells express wild-type p53, we therefore wanted to know whether sodium pump α3 promotes CRC metastasis *via* downregulation of p53-dependent pathways. The p53 downstream targets PTEN, IGFBP3, TSC2, AMPK1 and PhLDA3 were analyzed by qPCR. As shown in **Figure 2A**, silencing α3 in HCT-116 cells upregulated the transcript levels of PTEN and IGFBP3 but not the levels of TSC2, AMPK1 and PhLDA3, indicating that PTEN and IGFBP3 may be involved in α3-mediated metastasis of CRC. Reciprocally, overexpression of α3 in HT29 cells with low α3 expression downregulated the transcript levels of PTEN and IGFBP3 (**Figure 2B**). Consistent with these findings, silencing α3 increased p53 expression and that of its downstream targets PTEN and IGFBP3 at the protein level, which was followed by downregulation of the active form of mTOR and AKT and upregulation of the metastatic biomarker E-cadherin (**Figure 2C**); however, overexpression of α3 attenuated these actions (**Figure 2C**). Here, we further confirmed that p53 negatively regulated the PTEN/IGFBP3-mediated mTOR-E-cadherin pathway in colon cancer cells (**Figure 2D**). Furthermore, knockdown of p53 significantly attenuated the effect of silencing α3 on the protein expression of PTEN/IGFBP3/p-mTOR/p-AKT/E-cadherin and migration (**Figures 2E, F**). The mTOR inhibitors rapamycin and sapanisertib dramatically inhibited the migration of HCT-116 cells (**Figure 2G**). Together, α3 promotes metastasis of CRC *via* the p53-PTEN/IGFBP3-mTOR axis.

### Bufalin Inhibits α3-Mediated CRC Metastasis *via* the p53-PTEN/IGFBP3-mTOR Pathway

**Figure 3:**
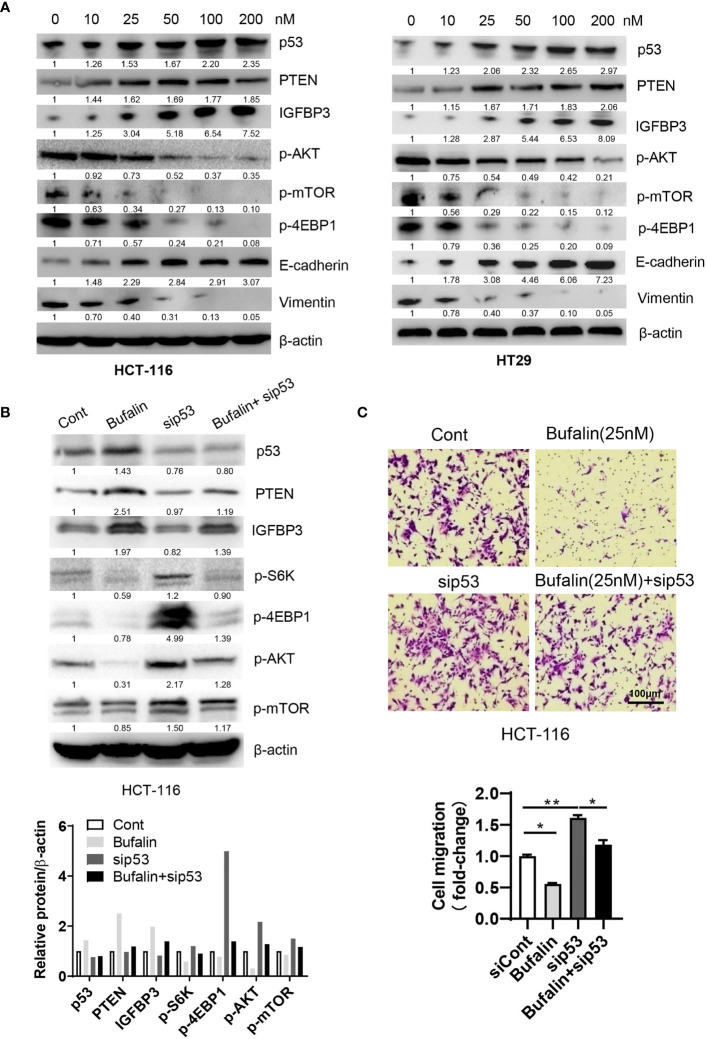
Bufalin inhibits the migration of CRC cells *via* the p53-PTEN/IGFBP3-mTOR axis. **(A)** HCT-116 or HT29 cells were treated with the sodium pump inhibitor bufalin (0, 10, 25, 50, 100, 200 nM) for 48 h and then harvested for Western blotting analysis. **(B, C)** HCT-116 cells were transfected with siRNAs targeting p53 in the presence or absence of bufalin (25 nM) for 48 h. A portion of cells were harvested for Western blotting analysis. The other portions were plated into Transwell plates overnight and then stained with crystal violet (0.5% w/v) for cell migration analysis. Scale bar, 100 μm. One-way ANOVA with Tukey’s multiple comparisons test was used. *p ≤ 0.05, **p ≤ 0.01.

To validate the mechanisms underlying the effects of sodium pump α3 on the migration of CRC cells, we subsequently examined the role of the sodium pump inhibitor bufalin on cell migration. We first observed that bufalin increased the expression of p53 and its targets PTEN and IGFBP3 and decreased the expression p-AKT (Ser473), and p-mTOR (Ser2448), which was followed by upregulation of E-cadherin and downregulation of vimentin in a dose-dependent manner (**Figure 3A**). We next determined whether the role of bufalin in CRC is dependent on p53. As shown in **Figure 3B**, silencing p53 partly blocked the bufalin-induced upregulation of PTEN and IGFBP3 expression and then promoted AKT and mTOR activation. Consistent with this finding, silencing p53 dramatically attenuated the bufalin-mediated inhibition of cell migration in HCT-116 cells (**Figure 3C**). These data demonstrated that bufalin could block the metastasis of CRC *via* the p53-dependent PTEN/IGFBP3-mTOR pathway.

### Knockdown of α3 or Targeting Sodium Pumps Protects Against Liver Metastasis of CRC *In Vivo*

**Figure 4:**
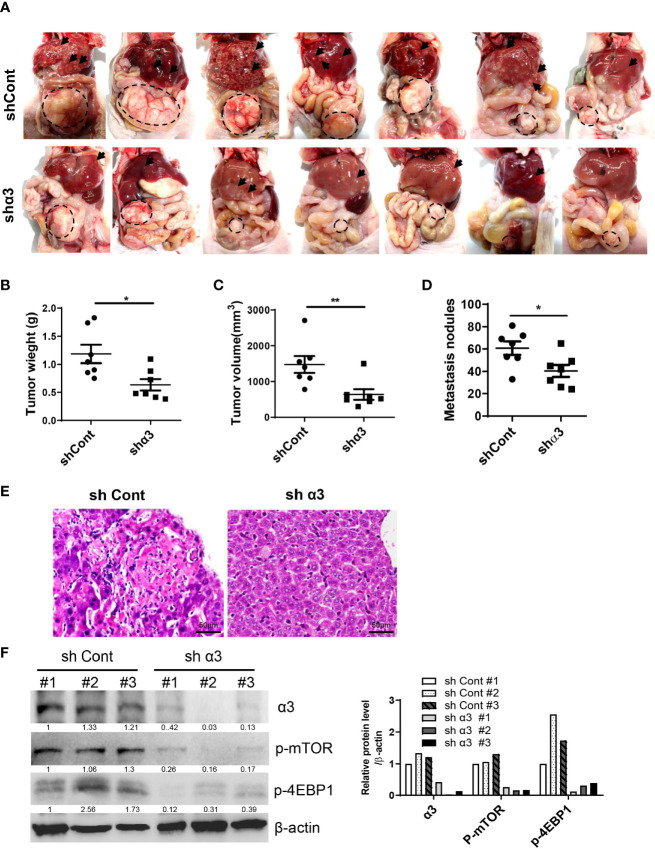
Knockdown of α3 protects against CRC liver metastasis *in vivo.* **(A–F)** An HCT-116 orthotopic xenograft mouse model was established using 8-week-old female mice. Mice were observed for 6 weeks and then euthanized. Knockdown of α3 decreased liver metastasis of HCT116 cells **(A)**. Arrows indicate metastatic nodules. Dashed lines delineate primary xenograft colon tumor grown in the cecum wall. The primary tumor weight and volume were quantified **(B, C)**. The nodule number on the liver surface was also counted **(D)**. n=7 for each group. Student t-test was used. *p < 0.05; **p < 0.01. The liver metastatic nodules were stained by H&E **(E)**. The scale bar represents 50 μm. The expression levels of α3, p-mTOR, and p-4EBP1 in liver tissues were analyzed by Western blotting **(F)**.

**Figure 5:**
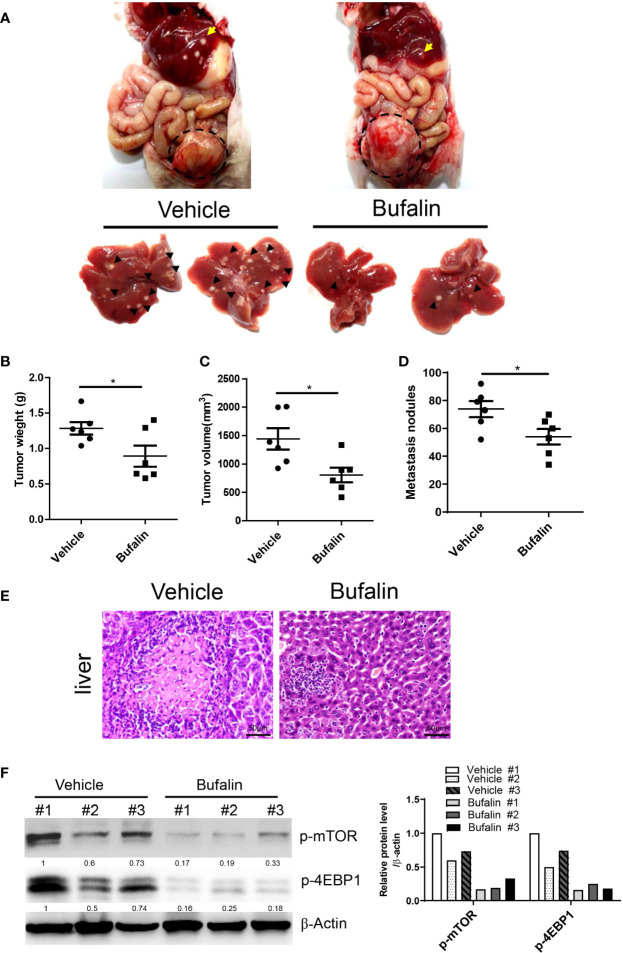
Bufalin inhibits α3-mediated CRC liver metastasis *in vivo.* **(A–F)** HCT-116 orthotopic xenograft mice were treated with bufalin (1.5 mg/kg) every other day for six weeks. Inhibition of the sodium pump by bufalin inhibited liver metastasis of HCT116 cells **(A)**. Arrows indicate metastatic nodules. Dashed lines delineate primary xenograft colon tumor grown in the cecum wall. The primary tumor weight and volume were quantified **(B, C)**. The tumor nodule number on the liver surface was counted **(D)**. n=6 for each group. Student t-test was used. *p < 0.05. The liver metastatic nodules were stained by H&E **(E)**. The scale bar represents 50 μm. The expression levels of p-mTOR and p-4EBP1 in liver tissues were analyzed by Western blotting **(F)**.

To determine the role of sodium pump α3 in CRC liver metastasis, we established an HCT-116 orthotopic xenograft mouse model. As shown in **Figure 4A**, representative images showed that knockdown of α3 could significantly block HCT-116 cell growth at cecum wall and metastasis to liver compared with the control group, as indicated by decreased primary tumor weight and volume and metastatic nodules in the α3-knockdown group (**Figures 4B–D**). The tissues were stained by H&E and revealed the decreased CRC liver metastasis in the α3-knockdown group (**Figure 4E**). Furthermore, the activity of mTOR was analyzed in tissue samples. Knockdown of α3 significantly decreased mTOR activity, as indicated by downregulation of p-mTOR and its target p-4EBP1 (**Figure 4F**). To validate these findings, we determined the effect of the sodium pump inhibitor bufalin on HCT-116 cell liver metastasis. We found that bufalin significantly inhibited CRC cell growth and the metastasis of the tumor from the cecum to the liver, as indicated by the decrease of tumor weight, tumor volume, and metastatic nodules on liver after bufalin treatment (**Figures 5A–E**). In addition, consistent with the knockdown effect of α3, bufalin reduced the expression of p-mTOR and p-4EBP1 (**Figure 5F**). Taken together, interfering with or targeting α3 protects against CRC liver metastasis.

### Correlation Between α3 Expression and mTOR Activity in CRC Tissues

**Figure 6:**
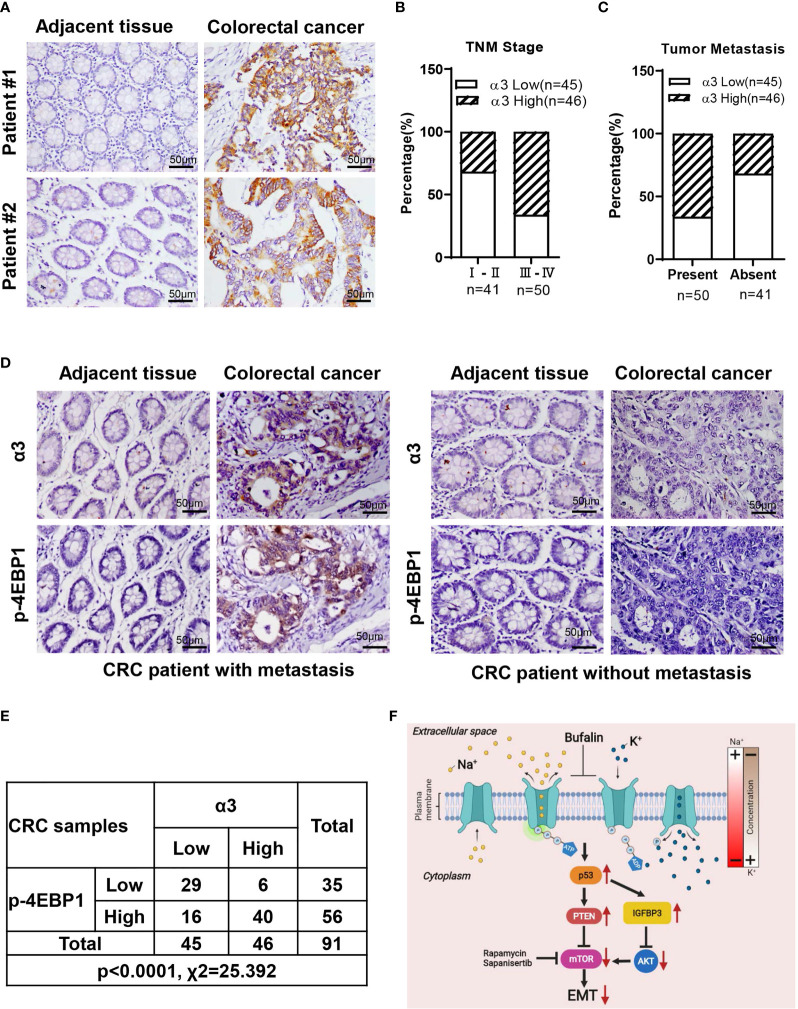
Correlation between α3 and mTOR activity in 91 CRC tissues. **(A)** Representative images of IHC staining for α3 in CRC tissues and matched adjacent normal tissues. **(B, C)** The relationships between α3 and TNM stage **(B)** and metastasis **(C)** were determined. **(D, E)** The correlation between α3 and p-4EBP1 was evaluated in 91 CRC tissues and matched adjacent normal tissues. **(F)** A model of CRC cell metastasis driven by elevated α3 expression *via* the p53-PTEN/IGFBP3-mTOR axis, which is blocked by bufalin. Scale bar, 50 μm.

To determine whether α3 is related to CRC, we analyzed the relationship between α3 expression and clinicopathologic characteristics in 91 CRC patients. The results indicated that α3 was highly expressed in 58.24% (53/91) of tumor tissues, and its expression was positively associated with TNM stage and metastasis of CRC patients (**Figures 6A–C** and **Table 1**). Furthermore, we used IHC staining to measure α3 expression in CRC tissues and correlated this staining with p-4EBP1 staining in the same 91 CRC tissues (**Figure 6D**). We found that α3 and p4EBP1were stronger in CRC tumors with metastasis than without metastasis. In total, 43.96% (40/91) of CRC tissues exhibited α3-positive and p4EBP1-positive staining, which was statistically significantly different compared to the staining rate in the adjacent normal tissues, suggesting possible mTOR activation by α3 in CRC (**Figure 6E**). Taken together, our results demonstrated that elevated expression of α3 in the CRC of mice or patients promoted metastasis *via* downregulation of p53-PTEN/IGFBP3 and subsequent activation of mTOR, and bufalin inhibited this action (**Figure 6F**).

**Table 1:** Relationships between α3 protein expression and clinicopathologic characteristics in 91 CRC patients.

Variable	Cases	Low α3	High α3	p Value
Age(years)				0.59
<55	24	13	11	
≥55	67	32	35	
Sex				0.116
Female	45	26	19	
Male	46	19	27	
TNM Stage				0.001***
I-II	41	28	13	
III-IV	50	17	33	
Histologic Grade				0.6
G1G2	63	30	33	
G3	28	15	13	
Metastasis				0.001***
Present	50	17	33	
Absent	41	28	13	

Calculated using the χ^2^ test. ***p ≤ 0.001 were considered statistically significant. Bolded value indicated statistically significant.

## Discussion

Colorectal carcinoma is the 3rd most common morbidity and the 4th leading cause of cancer-related death in the world (35). Every year, approximately 1.2 million cases of colorectal carcinoma (CRC) are newly diagnosed worldwide (36). Metastasis is the process by which tumor cells spread from the original site to the secondary sites, resulting in increased mortality in CRC (37, 38). The unclear mechanisms of CRC metastasis inhibit the development of treatment and prevention strategies. Recent studies have reported that the sodium pump α3 subunit exhibits increased expression in CRC and is associated with liver metastasis (8), but the underlying mechanism is not yet known.

There are 50-60% CRC patients with p53 mutation (39). p53 plays a critical role in the regulation of cell migration (34, 34, 38). Our study also demonstrated that p53 negatively regulated cell migration in CRC cells. p53 loss or mutation activates mTOR *via* a reduction in PTEN accumulation and AKT activation in mouse liver tumorigenesis (40). Consistent with this finding, we found that p53 downregulated mTOR activity *via* PTEN/IGFBP3 in CRC cells. Furthermore, we also found that the expression of p53 is negatively associated with the expression of sodium pump α3. This finding is consistent with a previous report that the sodium pump inhibitor bufalin upregulated p53 expression in 40-50% CRC patients with wild-type p53 (41, 42).

Cardiac glycosides such as digoxin and bufalin, which are used to treat heart diseases, have been demonstrated to kill various cancers. Recent studies indicated that cardiac glycosides were more susceptible to inhibiting sodium pump α3 subunit activity than any other subunit (43). Here, we found that bufalin could significantly inhibit cell migration in HCT-116 cells with high α3 expression, suggesting that bufalin may be a potential drug for CRC cell metastasis. Regarding the anticancer mechanisms of bufalin in CRC, our previous studies demonstrated that bufalin induced cell cycle arrest at prometaphase and cell death *via* autophagy or apoptosis (23, 44). In addition, our previous study indicated that sodium pump a3 and its inhibitor bufalin regulated CRC cell proliferation *via* PI3K-Akt-Aurora A/B pathway (45). Here, we reported that bufalin inhibited CRC liver metastasis *via* the p53-PTEN/IGFBP3 axis, which enriched our current understanding of the mechanism of bufalin in CRC. As IGFBP3, a well known protein, negatively regulates Akt activation (26, 27), it is possible that bufalin may regulate CRC cell proliferation *via* p53-IGFBP3-Akt-Aurora A/B pathway.

Previous studies demonstrated that sodium pumps are associated with cell metastasis in CRC (8), lung cancer (15), and breast cancer (16). Sodium pump-mediated metastasis remains unclear. Here, we reported that sodium pump α3 promoted CRC liver metastasis *via* p53-PTEN/IGFBP3-mTOR. However, there are 2 major issues worthy of further investigation: 1) how α3 regulates p53 expression and 2) how activated mTOR promotes metastasis and regulates EMT.

In conclusion, the sodium pump α3 subunit is highly expressed in CRC tissues and positively associated with poor prognosis in CRC patients. α3 promotes CRC liver metastasis *via* the p53-PTEN/IGFBP3-mTOR axis, and the sodium pump inhibitor bufalin can inhibit this action. α3 is a poor prognostic marker of CRC and/or a potential therapeutic target for CRC patients with wild type p53.

## Data Availability Statement

The original contributions presented in the study are included in the article/**Supplementary Material**. Further inquiries can be directed to the corresponding author.

## Ethics Statement

The studies involving human participants were reviewed and approved by the Ethics Committee of Affiliated Hospital of Jiangnan University. The patients/participants provided their written informed consent to participate in this study.

## Author Contributions

C-MX conceived and supervised this project. C-MX designed and developed the hypothesis. DW, H-JX, X-TL, JZ, and WW performed cell experiments, reverse transcription PCR, Western blotting, and C-MX and DW analysed the data. H-QY and Y-JZ performed animal study, and C-MX analysed the data. H-QY performed histological staining, immunohistochemistry, and analysed the data. TW and X-YL collected the CRC tissues and clinical information. C-MX and DW wrote the manuscript. Final approval of manuscript: All authors.

## Funding

This work was supported by Natural Science Foundation of Chongqing (cstc2019jcyj-msxmX0519) to C-MX and National Natural Science Foundation of China (82003108) to X-YL.

## Conflict of Interest

The authors declare that the research was conducted in the absence of any commercial or financial relationships that could be construed as a potential conflict of interest.

## Publisher’s Note

All claims expressed in this article are solely those of the authors and do not necessarily represent those of their affiliated organizations, or those of the publisher, the editors and the reviewers. Any product that may be evaluated in this article, or claim that may be made by its manufacturer, is not guaranteed or endorsed by the publisher.
